# RADIX: rhizoslide platform allowing high throughput digital image analysis of root system expansion

**DOI:** 10.1186/s13007-016-0140-8

**Published:** 2016-09-05

**Authors:** Chantal Le Marié, Norbert Kirchgessner, Patrick Flütsch, Johannes Pfeifer, Achim Walter, Andreas Hund

**Affiliations:** Institute of Agricultural Sciences, ETH Zurich, Universitätstrasse 2, 8092 Zurich, Switzerland

**Keywords:** Abiotic stress, Corn, Maize, Rhizotron, Root foraging, Split root, Root–shoot interaction, Quantitative trait loci, Imaging

## Abstract

**Background:**

Phenotyping of genotype-by-environment interactions in the root-zone is of major importance for crop improvement as the spatial distribution of a plant’s root system is crucial for a plant to access water and nutrient resources of the soil. However, so far it is unclear to what extent genetic variations in root system responses to spatially varying soil resources can be utilized for breeding applications. Among others, one limiting factor is the absence of phenotyping platforms allowing the analysis of such interactions.

**Results:**

We developed a system that is able to (a) monitor root and shoot growth synchronously, (b) investigate their dynamic responses and (c) analyse the effect of heterogeneous N distribution to parts of the root system in a split-nutrient setup with a throughput (200 individual maize plants at once) sufficient for mapping of quantitative trait loci or for screens of multiple environmental factors. In a test trial, 24 maize genotypes were grown under split nitrogen conditions and the response of shoot and root growth was investigated. An almost double elongation rate of crown and lateral roots was observed under high N for all genotypes. The intensity of genotype-specific responses varied strongly. For example, elongation of crown roots differed almost two times between the fastest and slowest growing genotype. A stronger selective root placement in the high-N compartment was related to an increased shoot development indicating that early vigour might be related to a more intense foraging behaviour.

**Conclusion:**

To our knowledge, RADIX is the only system currently existing which allows studying the differential response of crown roots to split-nutrient application to quantify foraging behaviour in genome mapping or selection experiments. In doing so, changes in root and shoot development and the connection to plant performance can be investigated.

**Electronic supplementary material:**

The online version of this article (doi:10.1186/s13007-016-0140-8) contains supplementary material, which is available to authorized users.

## Background

The spatial distribution of a plant’s root system is crucial for the success of the plant in a given environment because it determines how easily a plant can access water and nutrient resources of the soil. Root system architecture (RSA) is governed by the environment, but also by the genotype. Today it is unclear to what extent genetic variations in root system responses to spatially varying soil resources can be utilized. Closing this knowledge gap is certainly one step forward towards improving resource-use efficient crops. First studies provided evidence for an advantage of certain root architectural characteristics, such as deep rooting under drought [1, 2], shallow rooting under low phosphorus availability [3] or steep rooting angles under low nitrogen availability [4–7].

One major problem studying root growth is the fact that roots are often hidden in their growth matrix like soil unless the growth medium is transparent such as a gel. In the field, even the most advanced procedures offer only a partial glimpse of RSA. To overcome this limitation, research was done on developing monitoring systems of root growth in greenhouse approaches that provide visibility of the roots. Various phenotyping platforms were developed to monitor root growth non-invasively in soil based systems such as rhizotrons/-boxes containing sand/soil substrates [8], via X-ray micro-tomography [9, 10] or magnetic resonance imaging [11, 12] measurements. However, to date the throughput of these techniques is still not high enough for the assessment of large sets of genotypes as needed for mapping studies of quantitative trait loci (QTL).

For fast and simple screening, germination paper has become state-of-the art in many labs focusing on roots [13–16]. The systems using filter paper as substrate are sometimes called “growth pouches” [17]. Rhizoslides are a large-scale refinement of growth pouches, which allow to study root development for a prolonged period of time [18]. The growth pouch system was developed to map QTLs in collections of about 200 genotypes in various populations of maize during the very early seedling development [19–22] whereas the rhizoslides allow studying root growth until four-leaf stage.

A limitation of the growth pouches was that only the dynamics of the embryonic roots could be studied, ignoring the most prominent root type of maize, the shoot born crown roots. Albeit embryonic roots and crown roots are under different genetic control [23, 24] and may respond distinctly different to environmental stimuli. For example, Yu et al. [25], reviewed that embryonic roots of maize seedlings respond to nitrogen-rich patches by increasing the length of lateral roots, while crown roots of adult plants increased length and density of lateral roots [26, 27].

It is widely accepted that plants can adjust to localized soil enrichment by physiological changes [28]. Yet, to date there is still a lack of understanding the utility of this response for plant improvement because of a lack of appropriate methods to quantify responses of different genotypes. For breeding research, it would be highly beneficial to select genotypes not only by their final phenotype but also by their dynamic response to temporally changing or spatially varying nutrient availability. For example, Lynch et al. [7] proposed that an “optimal” root system should ignore local resource availability in the top soil and should grow into deep soil layers to be prepared against drought and nitrogen (N) stress later in the season. Thus, a reduced response to spatial variability would be desirable. Others propose the opposite, i.e. that a better response to local variation of nutrients would be beneficial to take advantage of side banded fertilizer placements [27]. A proof-of-concept of the split-nutrient application in rhizoslides was presented by in t’ Zandt et al. [29] observing a strong differential response of crown roots to N. It is conceivable that there is an optimum response to N, which may vary depending on the species, crop management and target environment. With phenotyping systems, such as rhizoslides, it will be possible to test corresponding research hypotheses in this context. One opportunity to evaluate plant performance is to study the shoot development. The measurement of canopy development is an important component of sound root research as the shoot acts as source of carbohydrates and sink for nutrient and water. There is a close link between root and shoot growth in many crops (see [30] for a discussion). Hence, a better understanding of the relationship between root and shoot development is an important need in order to identify heritable root traits for which the additional costs of direct selection are justified as pointed out by Wissuwa et al. [31]. For example, by studying the development of rooting depth in relation to shoot development in a diverse set of maize, Grieder et al. [32] found a strong linear relationship between the two traits under well watered conditions, leaving little genetic variation to alter rooting depth without changing canopy size. The non-destructive measurement of canopy size is well established [33, 34] and the adaptation of linear models is an accepted method [35] whereas for root traits dynamic studies were not often performed and less is known about models fitting this need. Pioneer work was done by Adu et al. [13] modelling the root growth dynamics of *Brassica rapa.*

With the rhizoslides, a second generation of the growth pouch system was developed [18] and tested for its suitability for split-nutrient application (in t’ Zandt et al. [29]). Here we present the RADIX platform facilitating an improved irrigation, handling and imaging of rhizoslides. The current version contains 100 slides and enables the cultivation of 200 maize plants at the same time, one on each side of the slide. The aim of the study was to demonstrate the value of the platform for phenotyping of natural variation of root traits in maize by investigating (a) root and shoot growth beyond the early seedling stage, and (b) the effect of split-nutrient application on the crown root development. This setup should provide information on genotypic variability of N uptake under spatially heterogeneous N availability.

## Methods

### Cultivation in the RADIX platform

**Fig. 1:**
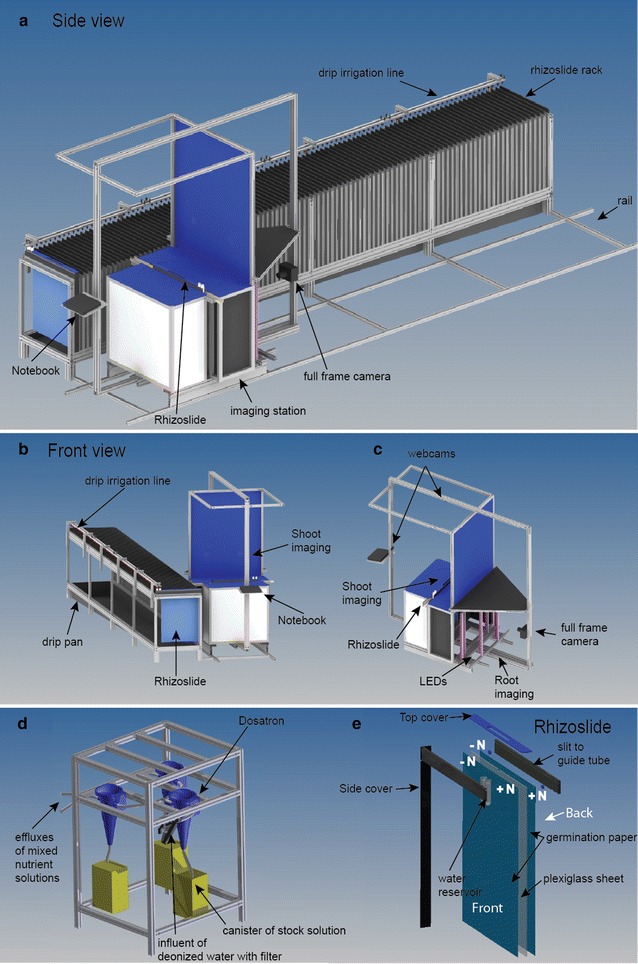
**a**, **b** Schematic figures of the RADIX platform with the imaging station moving on rails along a rack holding the rhizoslides. After positioning the station, the rhizoslides can be slid onto the station. Two drip irrigation lines are mounted at the backside of the rack enabling the supply of two different nutrient concentrations. **c** The imaging station holds two webcams to image the shoot from the side and from the top; a DSLR camera is mounted at 1 m distance from the slide surface to monitor the roots. Roots are illuminated by LED bars (three on each side); illumination is controlled by a microcontroller built and programmed in Arduino 1.0. Rhizoslides on the slide mount can be turned by 180° to image both sides of the rhizoslide. **d** The dosatron pump system consists of one dosatron supplying base nutrient solution that is split up to two dosatrons allowing for differential nutrient supply. **e** Scheme of a rhizoslide and the irrigation for the split root setup. High and low N concentrations were dripped into reservoirs from where tubes transported the nutrient solution to the split-nutrient germination paper separated by the wax barrier

Plants were grown in so called “rhizoslides” that consist of two PVC bars (600 × 60 × 10 mm) and a plexiglass sheet (650 × 530 × 4 mm) fixed with two screws between the bars (Fig. 1e) [18]. The dimensions of the assembled rhizoslide were 600 × 60 × 38 mm. The plexiglass sheet was covered with wet germination paper (49 × 61 cm) (Anchor Steel Blue Seed Germination Blotter, Anchor Papers Co, USA) on both sides, serving as substrate. These were in turn covered by a transparent, oriented polypropylene (OPP) foil with micro holes of 70 ± 10 µm with a distance of 105 mm (in x and y orientation) to allow for gas exchange (Maag, GmbH, Iserlohn, Germany).

For sterilization, the germination paper was heated in three cycles from room temperature to 80 °C and kept at this temperature for at least 120 min. Between the heating periods the paper was kept for 20–22 h in an oven at 37 °C and 50 % relative humidity [36]. To prevent fungal growth during the experiment, a method described by Bohn et al. [37] was used. The germination paper was moistened with deionized water containing 2.5 gL^−1^ Malvin (Syngenta Agro AG, Dielsdorf, Switzerland) containing the active component Captan. Once a week, each rhizoslide was watered with 15 mL of the Malvin solution to balance for leached fungicide.

The plant material consisted of the core EURoot maize panel (www.euroot.eu), with 25 dent inbred lines B73 (1), EC169 (4), EZ11A (13), F98902 (5), FV353 (6), LH38 (14), MS153 (7), Oh33 (16), EZ37 (10), MS71 (22), Os420 RootABA− (11), Os420 RootABA+ (12), Mo17 (3), FC1890 (18), LAN496 (23), W64A (20), F7028 (8), EZ47 (9), B100 (24), N6 (25), N25 (26), A347 (32), PH207 (33), F1808 (34), B97 (36),) crossed to UH007 as common flint tester supplied by Delley seeds and plants Ltd. (DSP Ltd., Switzerland). The number in brackets indicated the EU_ID.

Seeds were surface sterilized with 0.3 % PrevicurN (Bayer CropScience GmbH, Monheim, Germany) for 30 min and became touch dry without rinsing the seed. Subsequently, seeds were incubated in Petri dishes lined on paper soaked with a soil bacteria mixture (0.0001 % RhizoPlus 42, Andermatt Biocontrol AG, Grossdietwil, Switzerland and 0.01 % FZB24, Bayer AG CropScience, Zollikofen, Switzerland) to promote the development of a healthy rhizosphere and to prevent seed-borne infections. Seeds were kept for 48 h at 26 °C in the dark for germination and were then transferred into the rhizoslides.

For this study, 100 Rhizoslides were prepared with one plant in each rhizoslide. The seed was placed on top of the germination paper enabling simultaneous study of seminal and crown roots (for details, see [24]). A split-nutrient system was created by ironing a vertical water impermeable wax barrier into the germination paper and by a drip irrigation system (for details, see [29]). The roots could freely develop on both germination papers and on both sides of the wax barrier. Each paper was a split nutrient setup; irrigated with a high and low N solution, respectively. All 100 rhizoslides were supplied with a solution with low N (10 % of full strength ~ high N solution) for 14 days (2–3 leaf stage). Subsequently, one side was irrigated with a thousand times lower concentration of N compared to the other side of the wax barrier (Fig. 1e). The split root treatment remained for 14 days until the end of the experiment (3–4 leaf stage). For studying root growth, images were taken every second day.

### Composition of the nutrient solutions

To prevent precipitation, two stock solutions were prepared. The solution A was identical for both treatments containing: KH_2_PO_4_: 0.5 mmol L^−1^; MgSO_4_ × 7 H_2_O: 2 mmol L^−1^; MnCl_2_ × 4 H_2_O: 9.15 µmol L^−1^; CuSO_4_ × 5 H_2_O: 0.2 µmol L^−1^; H_3_BO_3_: 46.25 µmol L^−1^; Na_2_MoO_4_ × 2 H_2_O: 0.58 µmol L^−1^; ZnSO_4_ × 7 H_2_O: 0.77 µmol L^−1^; FeEDTA (C_10_H_12_FeN_2_NaO_8_ × H_2_O): 0.04 mmol L^−1^, (pH ~ 6). The solution B contained: KNO_3_, Ca(NO_3_)_2_ × 4H_2_O and NH_4_NO_3_, (pH ~ 6). The final concentrations in the high N treatment were: KNO_3_: 4.5 mmol L^−1^; Ca(NO_3_)_2_ × 4H_2_O: 4 mmol L^−1^; NH_4_NO_3_: 1 mmol L^−1^. In the low N treatment, the ammonium and nitrate concentrations were reduced. The solution contained: KNO_3_: 5 µmol L^−1^; Ca(NO_3_)_2_ × 4 H_2_O: 5 µmol L^−1^; NH_4_NO_3_: 1 µmol L^−1^and to balance the ion activities additionally KCl: 5 µmol L^−1^ and CaCl_2_ × 2H_2_O: 5 µmol L^−1^. The solutions were calculated using GEOCHEM-EZ [38]. pH was adjusted by adding KOH.

### Construction of the RADIX platform

The rhizoslides were hanging in a rack (500 × 65 × 80 cm) constructed with aluminium profiles (KANYA AG, Rüti, Schweiz) with 100 rhizoslide compartments. Each compartment was 60 cm long and 4 cm wide (Fig. 1a, Additional file 1). The distance between two neighbouring plants was 5 cm. A declining drip pan was built under the rack to collect the dripping nutrient solution (Fig. 1b) and a pump with automatic water level sensors (Gardena Comfort Tauchpumpe 9000 Aquasenor, Gardena Ulm, Germany) was used to pump down the solution. The sides of the rack were covered with a black polyethylene foil to prevent the roots against incidence of light. Each rhizoslide was covered on top with a cover plate containing holes for the shoot.

The irrigation system of every rhizoslide consisted of two PVC tubes (5/3 mm) (GVZ-Gossart AG, Otelfingen, Switzerland) and 25 mL PE tubes (Semadeni AG, Ostermundigen, Switzerland) acting as water/nutrient solution reservoirs. The reservoirs were filled via an irrigation system consisting of three Dosatron pumps (Diaphragm range 2.5 m^3^ h^−1^) (Dosatron, International S.A.S, Tresses, France) to mix the stock solutions (100 times higher than final concentration) with deionized water. The stock solutions were homogenized by circulation starting ½ h before the mixing of the solutions. One Dosatron was placed in series with two parallel switched Dosatrons (Fig. 1d).

The first Dosatron in the line mixed the identical stock solution A. In the following, the line was separated and the parallel switched Dosatrons were adding solution B either with a high/or low N concentration. The two lines were supplying two different pipes, each containing 100 micro drips (Netafilm CNL-Tropfer Junior, GVZ-Rossat, Otelfingen, Schweiz); dripping into one of the two nutrient solution reservoirs with a flow of 25 ml min^−1^ (Fig. 1a, b). Irrigation was controlled by an irrigation computer (Gardena, C1060 Profi, Ulm, Germany). The irrigation cycle was 4 h with duration of one minute. The amounts to 25 mL per micro drip summing up to a daily rate of 300 mL per slide.

The RADIX platform was placed in a cultivation room (720 × 330 × 250 cm). Environmental settings were a day period of 14 h light, a temperature of 28/24 °C (day/night) at seed level, approximately 50 % air humidity and a light intensity of 600 µmol m^−2^ s^−1^ photosynthetic active radiation at plant canopy level supplied by GreenPower LED production modules (Koninklijke Philips Electronics N.V., Amsterdam, Netherlands). Sixteen LED modules were arranged in four subunits with four modules each. The subunits were hanging from the ceiling and were equipped with a motor to adjust the distance of LED lamps and plant canopy level. Each subunit was controlled separately. The dimension of the lighting system was 500 × 70 cm and each subunit was 125 × 70 cm.

### Imaging

**Fig. 2:**
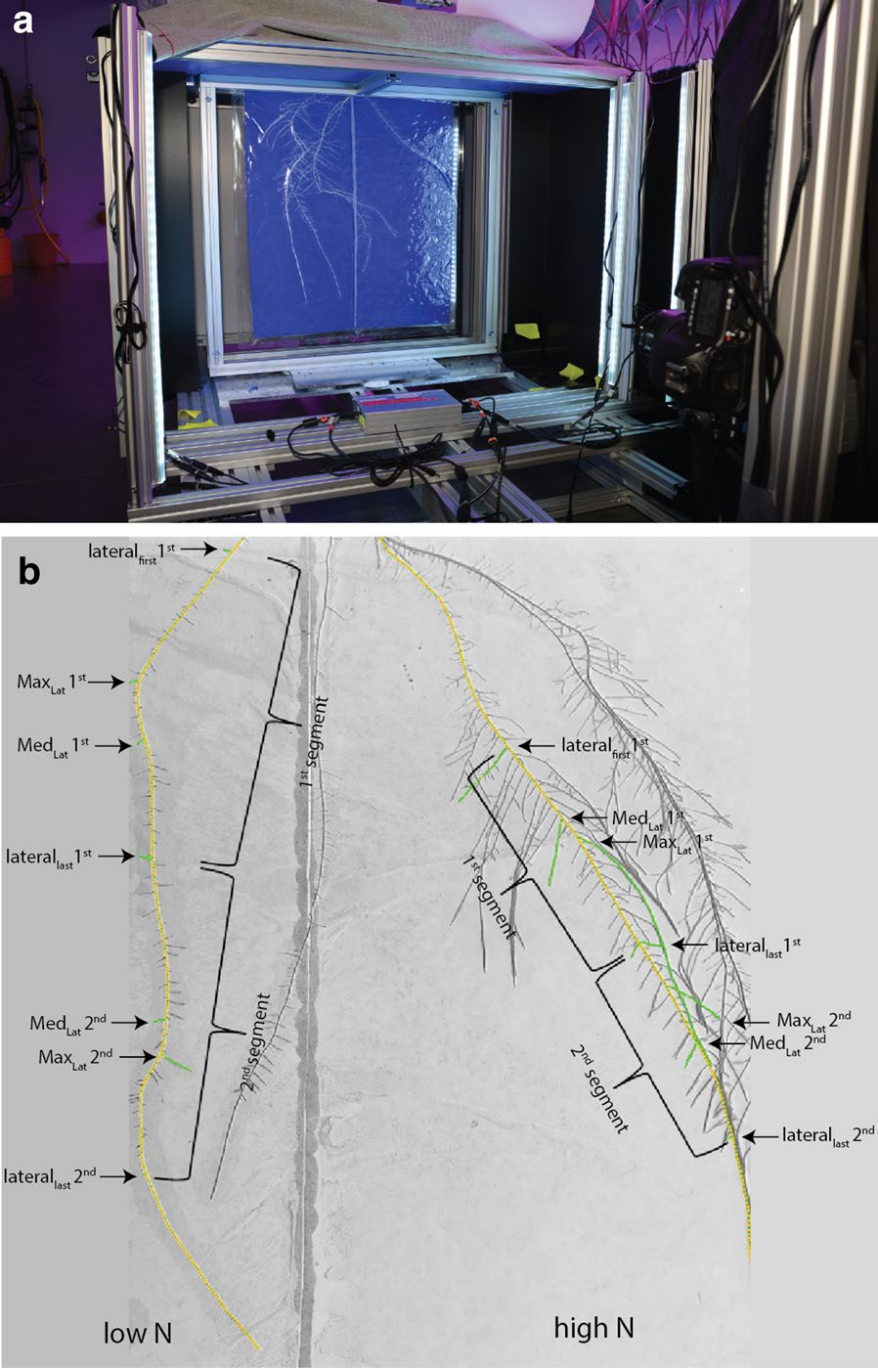
**a** Imaging car subunit which facilitated analysis of the root system. The root system on a split-root rhizoslide was placed approx. in 1 m distance to the 24 megapixel DSLR-camera. LED bars were placed between camera and rhizoslide on the* right* and* left side*, respectively. **b** Root system developed on one side of the rhizoslide and treated with a split-nitrogen application 10 days after solution change. Roots highlighted in* yellow* are the crown roots which were used in this example image to investigate crown root growth.* Waved brackets* indicate the two segments used to investigate the number of lateral roots and the medium (Med_Lat_) and longest (Max_Lat_) lateral root length in the first (Med_Lat_ 1st, Max_Lat_ 1st) and in the second (Med_Lat_ 2nd, Max_Lat_ 2nd) segment. The first segment started at the position of the the last developed lateral root (lateral_first_1st) at solution change and the second segment continued after the first segment (lateral_last_1st). The lateral roots used to investigate the medium and longest lateral root length in the two segments are highlighted in* green*

An imaging station (~168 × 164 × 110 cm) was constructed allowing for imaging the root system and the shoot at once (Fig. 1c, Additional file 2). The imaging station was moving on a rail parallel to the slide rack and the rhizoslides were manually slid onto the imaging mount (Fig. 1a, b). The mount was rotatable allowing imaging the root system growing on both sides of the slides with only one camera (Fig. 1c, Additional file 2). The camera was fixed at a distance of 1 m from the center of the acrylic sheet (Figs. 1c, 2a, Additional file 2). For shoot imaging, two webcams (Logitech, Business) were fixed at a distance of 65 cm (increased to 80 cm in the meantime in front of the rhizoslide) and 65 cm (increased to 115 cm) on top of the imaging car (Fig. 1c). The backside of the rhizoslide mount was covered with a blue Komatex plate for an optimal contrast between shoot and background (Fig. 1c). The root zone was hanging in a box constructed with black and white Komatex plates (Röhm AG, Schweiz) at the sides to prevent light scattering in the root zone; white plates in the back of the rhizoslide and black plates in front of it. The white plates in the back area were built as a light box, reflecting the light, whereas the black plates in the front inhibited reflections into the camera focus area (Figs. 1c, 2a). Only the front view was uncovered for root imaging. Black cloth was used to cover the area between camera and box (the cloth is not shown in Figs. 1c, 2a, Additional file 2).

Images were taken with a 22.3 mega pixel full-frame digital single-lens reflex camera (EOS 5D Mark III, Canon, Tokyo, Japan) equipped with a 50 mm lens (compact macro 50 mm f/2.5, Canon, Tokyo, Japan). The resolution of the images was around 0.13 mm pixel^−1^ (for details, see [18]). The flash lights were replaced by six LED bars (TRENOVA -Highpower- LED, PUR-LED GmbH & Co. KG, Selzen, Germany) allowing for a more homogenous illumination (Figs. 1a–c, 2a). Shoots were imaged with the two webcams simultaneously with the root system; one image from the front (maximal extent of the rhizoslide parallel to image plane) one image from the backside (180° turn on the horizontal axis) and images from the top view simultaneously with the side view images (in total four images). As the maximal extension of the leaf area differs between individual plants, imaging from different focal axis allows minimizing the error resulting from shoot orientation. In our setup, leaves were developing in parallel to the rhizoslide axis and therefore, imaging from the front and top rendered sufficient results and no additional imaging from the side was necessary.

### Image processing

The two root images taken with either illumination from the right or from the left side were combined to one 24 bit RGB image using Matlab (Version 7.12 The Mathworks, Natick, MA, USA) by keeping only the minimum tonal value present in each image (minimum tonal image) as described by Le Marié et al. [18].

In the current setup, the number of pixels of the front, back and top view of the maize shoot was used as proxy for canopy size. All four shoot images were segmented and used for leaf area calculation. Best results were obtained by calculating an average pixel number over the two sides and top views. Shoot images were segmented from the background using background subtraction and thresholding adaption to the image material written in Matlab R2013a (The Mathworks, Natick, MA, USA). Shoot segmentation was done in a two-step method on a feature map f: f = 2 * G-R-B. Segmenting with a high threshold 55 (in the result pixels of f with values ≥ 55 are set to 1 all others are set to 0) yields a coarse segmentation s_c_ and identifies all plant regions but does not nicely determine their borders. A fine segmentation s_f_ with Otsu’s method [39] resulted in more accurate sizes of the shoot regions but also yields additional image regions which were separated from the shoot. Therefore both segmentations were combined by selecting all regions of s_c_ and enlarging them to all therewith connected regions of s_f_.

### Calculation of the root growth rate

Crown roots of the first whorl were traced using SmartRoot [39]. Crown roots of the first whorl were not always placed on the high and low N side. Accordingly also roots of the second whorl were used. For tracing, one crown root was selected in a high N compartment and one crown root in a low N compartment, ideally with comparable developmental stages at the start of the split root treatment. Furthermore, only crown roots were selected that did not cross the wax barrier or reach the side or bottom of the germination paper at the end of the experiment. This step was necessary to ensure that zero growth in subsequent analyses was not due to the fact that a root reached the borders of the paper and to exclude compensatory growth of lateral roots. All selected roots were healthy and undamaged until the end of the experiment. Next, root elongation of crown roots over time was calculated using R (Foundation for Statistical Computing, Vienna, Austria). Single plants did not establish traceable crown roots on both sides of the split-nutrient germination paper as sometimes crown roots on one side reached the sides or bottom of the rhizoslide before the end of the experiment or no crown roots were formed on one of the split root sides. Though, the root system (seminal and crown roots) developed over the whole germination paper and was exposed to inhomogeneous N distribution. Therefore, it was expected that plant response should be comparable to plants showing traceable crown root growth in both compartments.

### Modelling the dynamics of root and shoot elongation

For a better understanding of responses towards a heterogeneous environment, a linear model was fitted to describe changes in root and shoot growth dynamics over time.

A simple linear regression was fit to the length development of each individual crown axile root using a linear regression fit lm() in R [40]:

1 $$L_{Cr} (t) = a + bt$$

where *L*_*Cr*_ (*t*) is the crown root length as a function of time *t*, *a* the estimated crown axile root length at the start of the split-nitrogen application (y-axis) and b is the slope of the regression line. The shoot growth was calculated simultaneously to the crown root length.

### Lateral root length and number

To investigate the effect of genotype and treatment on lateral root growth and number, medium and maximal lateral root length were measured and maximal lateral root number was counted at the last day of the experiment in two branching zones (Fig. 2b). The branching zone, developing after solution change, was defined by the youngest, most distal lateral root at solution change (proximal end of the branching zone) and the youngest, most distal lateral root at harvest (distal end of the branching zone) (Fig. 2b) [41]. The length of the branching zone was measured using the ruler tool in Image J (SmartRoot) and divided into two sections of equal length (first and second branching zone). A medium sized lateral root and the longest lateral root were traced with SmartRoot in both segments and the number of formed lateral roots was counted (Fig. 2b).

### Chlorophyll meter (SPAD) measurements

Chlorophyll measurements (SPAD-502, Minolta Corporations, Ramsey, NJ, USA) were done over the experimental period to control for plant health and chlorophyll content. Measurements were done on the last fully developed leaf (usually leaf 4) of every plant measuring at the shoot base, middle and tip of the leaf and averaging these values.

### Harvest

After 26 days in total, plants were harvested. The shoot was cut off at the base and leaf area and fresh weight were determined. Leaf area was determined using a leaf-area meter (LI-COR 3100, LI-COR, Lincoln, NE, USA). In order to determine shoot dry weight, shoots were dried at 60 °C for 4 days. Crown and seminal roots were separately harvested depending on the side they were growing (high or low N treatment), bagged and dried at 60 °C for 4 days. Only single lateral roots were growing into the germination paper and could not be harvested. To ensure a correct classification of crown roots, all crown and seminal roots were marked at harvest and an image was taken to compare with the last image taken in the RADIX setup.

### CN measurement

After drying, shoots were ground using a swing mill (Retsch MM200, Retsch GmbH, Haan, Germany) with a frequency of 25 s^−1^ for one minute. N content of the shoots was measured with a CN analyser (Flash EA, 1112 series) [42]. As reference plant material Alfalfa was used.

### Verification of the split nutrient system

To verify the separation of the two sides, a white germination paper was soacked in bromocresol green and afterwards watered with a basic (NH_4_HPO_4_) and an acidic [Ca(NO_3_)_2_] solution. A webcam was installed to image the elution. After 48 h the bromocresol was washed out and the paper was sprayed with it again to visualize the pH gradient (Additional file 3).

### Statistics

The rhizoslide experiment was a complete randomized block design with four replications. Each experimental unit consisted of one rhizoslide containing one plant. Linear mixed models were calculated in ASREML-R [43] according to the following full model for root observation in split N application:

2 $$y_{ijkl} = \mu + G_{i} + N_{j} + GN_{ij} + R_{k} + S_{l} + GR_{ik} + \varepsilon_{ijkl}$$

where *Y*_*ijk*_ is the trait measurement (slope or intercept of crown root length, length lateral root, number of children), of the root of the ith genotype (G = 1, 2, 3 … 24) within the *jth* N treatment (N = HN, LN) in the rth replication (k = 1, 2, 3, 4) on the lth side of the slide (l = front or back), µ is the general mean, G_i_ is the effect of genotype, N_j_ is the effect of the N treatment, GN_ij_ is the genotype-treatment interaction, S_l_ is the effect of the side of the slide the observed root grew on (front or back), *R*_*k*_ is the effect of the *kth* replicate, GR_ik_ is the genotype-replication interaction identifying individual slides and *ε*_*ijkl*_ is the residual error. The genotype-replication interaction was considered as random whereas all other effects were considered as fixed factors. A different variance was adjusted for each treatment level. For traits representing only one measurement per slide (SPAD, dry weight, leaf area, pixel number, slope or intercept of shoot growth), the model was reduced by only keeping the genotype and replicate as fixed factors. For root traits (Elongation rate crown roots (ER_Cr_), length of crown roots at solution change (intercept; IC_Cr_), length of representative lateral root (Med_Lat_), maximal lateral root length (Max_Lat_), number of lateral roots (No_Lat_) and root dry weight (DW_R_) a square root transformation was performed to achieve normal distribution and no outlier were removed. Root traits that were not normally distributed (length branching zone, branching density in zone 1 or 2, start branching zone) were excluded from further analysis. For shoot traits neither a transformation nor removing of outlier was necessary to achieve normal distribution.

Best linear unbiased estimators (BLUPs) were used to investigate the phenotypic diversity in the set [44]. Tukey’s honestly significant differences (Tukey HSD) were used as post hoc test and calculated as:

3 $$TukeyHSD = q*\sqrt {\frac{MSE}{n}}$$

where *q* is the critical value according to the chosen significance level and degrees of freedom, *MSE* is the mean square error calculated from the average standard error of the difference (avsed) supplied by the predict function of ASReml-R and *n* is the number of treatment levels.

#### Heritability

For heritability estimations across genotypes the model described by Eq. (2) was used, but the genotype and the genotype-treatment interaction were set as random factors. To estimate the heritability depending on the nitrogen treatment applied to the root system, a heterogeneous variance model with regard to the nitrogen-effect and its interactions was fit in ASReml-R following the recommendations of Butler et al. [43].

Mean-based heritability was calculated for each root trait within each nitrogen level and for each shoot trait as follows:

4 $$H^{2} = \frac{{\sigma_{g}^{2} }}{{\sigma_{g}^{2} + \frac{{\sigma_{e}^{2} }}{r}}}$$

where $\sigma_{g}^{2}$ and $\sigma_{e}^{2}$ are the genotypic and residual error variance respectively obtained from the linear mixed model fitting (Eq. 2) and *r* is the number of replications.

## Results and discussion

### RADIX: high throughput analysis of root elongation and dynamics of root topology

The established RADIX platform, which is based on rhizoslides as individual monitoring units, enables a high-throughput screening of RSA and root topology dynamics. One major advantage compared to existing phenotyping platforms such as GROWSCREEN-Rhizo [8] is the visibility of all targeted roots and a sufficient contrast for image processing. No targeted roots are hidden in deeper soil layers like it is the case in rhizotrons and limitations due to contrast between soil and root are usually a minor problem. The advantage of soil-based systems like rhizotrons and CT is that they are closer to natural conditions [8, 10]. Another advantage of rhizoslides is the high achievable throughput. The definition of high throughput usually depends on the analysed traits. Root accessibility and the measurement of growth and topology are certainly the most rate-limiting aspects studying root elongation and dynamics. Here, we define high throughput as the ability to evaluate QTL or association mapping populations of at least 200 genotypes within a minimum of three independent replications within half a year. The imaging process in rhizoslides takes approximately 1 min per slide (front and back side). Image processing in SmartRoot is still the time-limiting step (approximately 80 h to mark axile roots and the segments of lateral roots for a time series of 1400 images) but delivers detailed information of the development of root topology. Adu et al. [13] developed a screening method with medium throughput based on germination paper (n = 24). A scanner unit is fixed in front of each paper with roots growing on it. With this setup, higher temporal resolutions can be realized. However, a mobile imaging unit as realized in the RADIX balances between temporal resolution and throughput. As the response of the root system to stimuli, such as N, happens within days rather than minutes [29], daily or half daily scans are considered sufficient. With the current setup of the RADIX, the number of genotypes that can be evaluated remains the bottleneck. The highest throughput realized to date to study root dynamics in existing platforms is achieved in hydro- or aeroponic setups, yet without the possibility of split-nutrient application. It remains to be elucidated, under which conditions and for which root traits one or the other phenotyping systems is most suitable.

To our knowledge, RADIX is the only system currently existing which allows studying root growth responses under heterogeneous nutrient availabilities with a throughput high enough for QTL and association studies. The importance of root system architecture for N-uptake is supported by several positive QTL collocations between the two traits [45, 46]. However, to date it is still unclear, if increased uptake efficiency, increased N utilization efficiency [47, 48] or an optimal housekeeping with internal N resources [49], is most promising for optimal plant performance [46]. For example, under high N, the genetic variation in nutrient uptake seems to be related to uptake efficiency, whereas at low N, it seems to be related to utilization efficiency [48]. The RADIX setup could shed light onto this topic by studying individual plants under control as well as under stress conditions. In doing so, changes in root and shoot development and the connection to plant performance can be investigated.

### Dynamics of shoot development: an imperative for sound root research

**Fig. 3:**
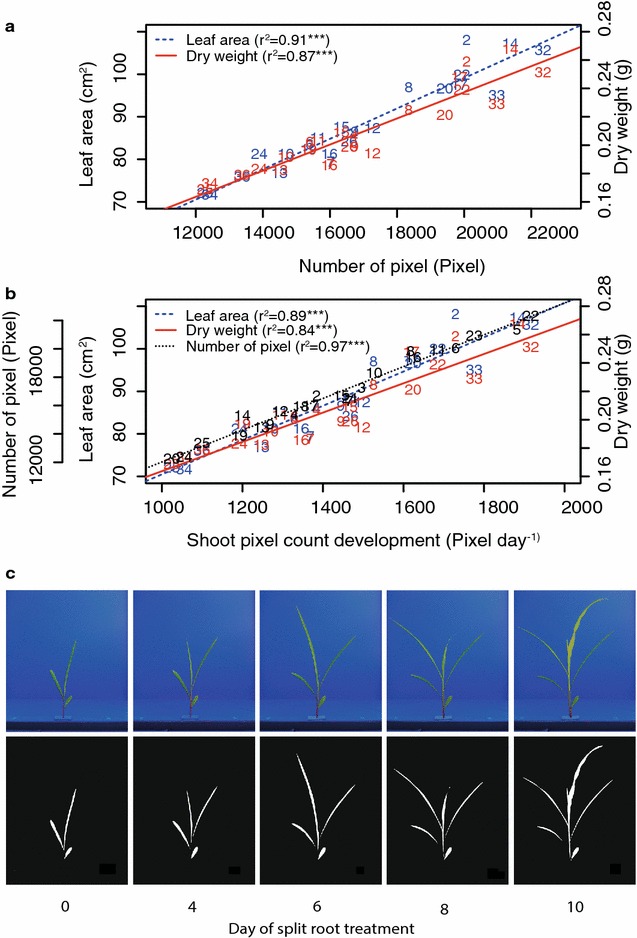
**a** Correlation between the average number of pixel (Pixel) of the* top* and* side* images and the leaf area (cm^−2^) or shoot dry weight (g) measured destructively at the end of experiment. **b** Correlation of shoot pixel count development (Pixel day^−1^) expressed as the slope best fitting the linear model and the number of pixels (Pixel), leaf area (cm^−2^) and shoot dry weight (g). **a**, **b** *Number*s represent the average of all replicates of a single genotype. For the corresponding genotypes see “Cultivation in the RADIX platform” in the “Methods” section. The standard error of the difference was 3392 pixels for the average number of pixel, 18.01 cm^−2^ for the leaf area, 0.05 g for the shoot dry weight and 315 pixels for the shoot pixel count development. **c** Growth series of an exemplary shoot and images of the segmented shoot

The platform was developed to allow assessing both root and shooting growth. In the current setup, the number of pixels of the front and back view of the maize shoot was used as proxy for canopy size. Shoots were successfully segmented from the background using a two-step method of coarse and fine segmentation (Fig. 3c). The number of pixels correlated well with leaf area r^2^  = 0.91***; Fig. 3a) as well as with shoot biomass (r^2^ = 0.87**; Fig. 3a). Best correlation could be achieved by calculating the mean value of the two images taken from the sides (rhizoslide parallel to camera focal axis in 0° (front) and 180° (back) and from the top (in total two front views and two top images). In a previous experiment, different imaging methods for the shoot were investigated. Best results were obtained by using the here presented combination of side and top view. This is in contradiction with investigations made in the GROWSCREEN-Rhizo facility where best results were obtained with images from the front side (0°) and a 90° horizontal rotation [8]. An explanation for this phenomenon could be the combination of the alternate leaf orientation in monocot species and a parallel orientation to the rhizoslide influenced by neighbouring plants (distance between two neighbouring plants of 5 cm) [45]. As leaves exceeded the imaging area 10 days after start of the treatment, the shoots dynamics could be assessed only up to this point. For future experiments, this was improved by constructing a larger vertical screen for the imaging station (already displayed in Fig. 1).

### Evaluation of shoot traits

**Fig. 4:**
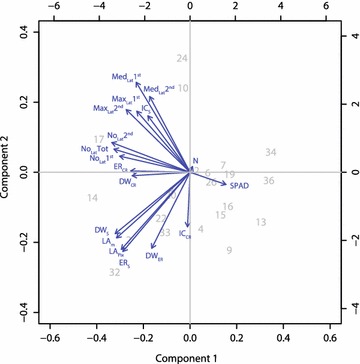
Principal component analysis (PCA) of traits based on best linear unbiased estimates (BLUPS). Abbreviations: Number of lateral roots in the first segment (No_Lat_ 1st) or second segment (No_Lat_ 2nd), length of representative lateral root in the first segment (Med_Lat_ 1st) or second segment (Med_Lat_ 2nd), maximal lateral root length in the first segment (Max_Lat_ 1st) or in the second segment (Max_Lat_ 2nd), total number of lateral roots (No_Lat_Tot), elongation rate crown roots (ER_Cr_), length of crown roots at solution change (intercept; IC_Cr_), embryonic root dry weight (DW_ER_), crown root dry weight (DW_CR_), leaf greenness (SPAD), leaf area measured (LA_m_), dry weight shoot (DW_S_), leaf area pixel based (LA_Pix_), shoot pixel count at solution change intercept (IC_S_), shoot pixel count development (ER_S_), N content in the leaf in % of total dry weight (N). The numbers in the PCA plot correspond to the EU_IDs. For the corresponding genotypes see “Cultivation in the RADIX platform” in the “Methods” section

Genotypes differed with respect to leaf area expansion (measured as pixel numbers), leaf area at harvest and shoot dry weight at harvest (Table 1a, b). For example, genotype A347 grew almost two times faster than genotype Oh33 (Additional file 4, Additional file 5B). As the traits, measured at final harvest, were strongly correlated with leaf area expansion, it can be concluded that final leaf area at this stage was driven by differences in vigour rather than differences in germination (Fig. 3b). Furthermore, the shoot pixel count at solution change was not correlated with the shoot pixel count development (Fig. 4, Additional file 6). No genotype-specific differences and no heritability were observed for chlorophyll content investigated by means of SPAD measurements (Table 1a, b). However, the genotypes differed in their N content (Table 1a). CN measurements revealed significant differences in N content between genotypes with a maximal N proportion of 5.07 % and a minimal proportion of 3.61 % (Table 1a, b). A high formation of biomass resulted in a reduction of leaf N content (Fig. 4) although this correlation was not significant (Additional file 6).

**Table 1:** (a) Results of the analysis of variance, (b) maximal, minimal and median values and heritability of traits

a	Root traits										Shoot traits						
	ER_Cr_	IC_Cr_	Med_Lat_		Max_Lat_		No_Lat_		DW_R_								
			1st	2nd	1st	2nd	1st	2nd	Embryonic	Crown	ER_Lf_	IC_S_	LA_Pix_	LA_m_	DW_S_	SPAD	N
	cm/d	cm	cm	cm	cm	cm	Counts	Counts	mg	mg	Pixel/d	Pixel	Pixel	cm^2^	g	Relative	%
Genotype	n.s.	n.s.	**	*	n.s.	***	***	***	n.s.	**	***	**	***	*	*	n.s.	**
Treatment	***	n.s.	***	***	***	***	***	***	***	***	NA	NA	NA	NA	NA	NA	NA
G:T	n.s.	n.s.	**	***	*	**	n.s.	n.s.	n.s.	***	NA	NA	NA	NA	NA	NA	NA

b	Treatment	Root traits										Shoot traits						
		ER_Cr_	IC_Cr_	Med_Lat_		Max_Lat_		No_Lat_		DW_R_ [mg]								
				1st	2nd	1st	2nd	1st	2nd	Embryonic	Crown	ER_Lf_	IC_S_	LA_Pix_	LA_m_	DW_S_	SPAD	N
		cm/d	cm	cm	cm	cm	cm	Counts	Counts	mg	mg	Pixel/d	Pixel	Pixel	cm^2^	g	Relative	%
Max	High	1.77	12.71	1.21	0.77	1.87	2.58	61.93	47.40	29.42	41.89	1914	1998	22,331	108.18	0.343	29.59	5.07
	Low	1.31	13.42	0.49	0.84	1.85	2.75	38.14	50.57	22.53	35.50							
Min	High	1.44	8.46	1.04	0.15	1.08	0.60	41.33	12.92	28.32	29.92	1023	527	12,261	71.75	0.117	29.59	3.61
	Low	1.06	9.17	0.32	0.24	1.06	0.71	17.54	16.37	20.79	15.79							
Median	High	1.65	11.12	1.09	0.47	1.45	1.72	50.56	28.90	28.73	34.63	1434	1301	16,556	86.4	0.192	29.59	4.30
	Low	1.16	11.83	0.37	0.46	1.48	1.77	26.77	33.30	21.42	22.10							
h^2^mean	High	0.41	0.03	0.72	0.61	0.41	0.69	0.53	0.57	0.07	0.15	0.58	0.53	0.59	0.45	0.46	0	0.52
	Low	0	0.03	0.26	0.42	0	0.59	0.34	0.30	0.19	0.57							

*ER*_*Cr*_ Elongation rate crown roots; *intercept*, *IC*_*Cs*_ length of crown roots at solution change; *Med*_*Lat*_ length of representative lateral root; *Max*_*Lat*_ maximal lateral root length; *No*_*Lat*_ number of lateral roots; *DW*_*R*_ root dry weight; *ER*_*S*_ shoot pixel count development; *intercept, IC*_*Cr*_ shoot pixel count at solution change; *LA*_*Pix*_ digital leaf area; *LA*_*m*_ measured leaf area; *DW*_*S*_ shoot dry weight; *SPAD* chlorophyll measurements, *N* content in the leaf in % of total dry weight. Significance level: *** ≤0.001; ** ≤0.01; * ≤0.05

### Crown root development

The elongation of crown axile roots was described best by linear models based on the observation of original data and residual plots (Additional files 7, 8, 9). In a previous study, best results were obtained by fitting a logistic model for the elongation rates of the crown roots on the high-N side and a regression model with segmented relationships for roots grown under no N [29]. The need for two different modelling approaches in the previous study did not enable the examination of interaction effects in a common linear mixed model. As already observed by in ’t Zandt et al. [29], crown root elongation tended to decrease in the low-N compartment (Additional file 8) while the elongation rate tended to increase in the high-N compartment (Additional file 7). However, the trend was far less pronounced in the present experiment enabling the fit of a common linear model (Additional file 9). The lower effects were possibly due to the replacement of the no-N treatment [29] by a low-N treatment. Unfortunately, the standard genotype UH007 × B73, used in the study of in ’t Zandt et al. [29], could not be investigated here, as it did not germinate.

First crown roots established on the germination paper after 2 weeks and their response to split-nutrient application could be measured for another 2 weeks. By then, they had reached the bottom or sides of the rhizoslides and the plants had reached four fully developed leaves. The realization of longer cultivation phases allowing the study of crown root dynamics is a major advantage compared to the growth pouch system used in past studies [17]. Genotypes differed in their developmental speed. While some genotypes started to form crown roots already seven days after start of the experiment, no crown roots were visible on the germination paper for others at the time point of solution change. The evaluation of the dynamic development of individual roots is certainly a way to account for such developmental differences.

For the genotypes F1808, EZ37, FC1890 and W64A only one or two replicates could be evaluated under low or high N as only those did develop crown roots until the time point of solution change (Additional files 7, 8). Twelve individual plants did not develop a traceable crown root on the high N side until the time point of solution change and seventeen on the low N side (Additional files 7, 8). We used a linear mixed model approach which handles missing values better than classical linear models [50]. One opportunity to increase the number of traceable crown roots would be to postpone the treatment to later developmental stages and another option would be to increase the number of replications.

Differences in the amount of roots growing at the low or high N side might influence the morphological responses of the roots to local high nitrate. However, in ‘t Zandt et al. [29] found no direct dependence of the dynamic of roots exposed to differential N supply in the rhizoslides concluding that the responses on either side occurred largely independent from each other.

### Genotype-by-nitrogen interaction for crown root development

The crown root elongation under low N was on average 26 % reduced compared to the high N side (Table 1b). The minimum observed elongation in the high N compartment was 1.44 cm d^−1^ and the maximal elongation was 1.77 cm d^−1^ (Table 1b). There were differences for the intercept, i.e. the crown root length at the time of solution change.

There was a significant genotype-by-treatment interaction for the response of crown root growth to split-nutrient application. More specifically, the crown axile roots at different N-concentrations showed genotype-dependent differential response. For example, crown roots of the genotype PH207 grew 39 % slower under low N conditions than under high N conditions whereas the genotype LAN 496 showed a much lower reduction in growth (15 %) under low N conditions (Table 1b, Additional file 7, 8). Interestingly, the heritability was high (h^2^ = 0.41) for roots grown under high N but zero for roots grown under low N conditions (Table 1b). This means that it is not possible to select genotypes based on their rooting behaviour on the low-N side of a split-nutrient setup. By contrast, the fast exploration of N rich patches and selective root placement is highly heritable and could be an advantageous trait with respect to inhomogeneity in the field or patch wise application of fertilizers e.g. by row fertilization. According to a model by Dunbabin et al. [51], selective root placement could lead to a more than two fold higher N uptake efficiency throughout the whole growing season, compared to a root system that is only poorly capable to respond to N. Already the study of in ’t Zandt et al. [29] demonstrated a oppositional response in the low N and high N compartment. However the study was limited to one genotype. To our knowledge, this is the first time that such a selective response could be observed for a larger set of maize genotypes.

### Length and density of lateral roots

Investigations of lateral root number and length were only done at the end of the experiment as in the study of in ’t Zandt [29] the maximal number of lateral roots in the segment was already reached two days after solution change in the high N compartment and after four days in the low N compartment. Thus, it was assumed that the final number of lateral roots is representative for the branching intensity in these segments. Furthermore, the number of laterals in the zone present before solution change did not significantly increase during the two weeks of treatment, hypothesizing that only newly developed root tissues are able to respond to N availability (Additional file 10).

In general, lateral roots became longer under high N compared to low N conditions (Table 1b, Additional file 5A-C). This finding is in close agreement with previous studies reporting a selective root placement within the high N compartment by stronger lateral root formation and growth [52, 53]. However, some genotypes were characterized by an opposed trend highlighting the opportunity to identify contrasting genotypes. Past studies already observed different responses for different species and even different genotypes within a species to the same environmental stimulus [54], but to date no study tried to use contrasting genotypic responses for mapping of quantitative traits.

The length of the branching zone (Tab 1. LBrZ) did not differ among genotypes but the number of lateral roots (Table 1, No_Lat_1st and No_Lat_2nd) in this zone was heritable and correlated with the axile root growth [r = 0.47* (No_Lat_1st) and r = 0.49* (No_Lat_2nd)]. Interestingly, the genotypes did not differ for the linear density of lateral roots per unit axile root length. Hence, the formation of lateral roots seemed to be mainly driven by a stronger axile root elongation rather than a higher branching density as no significant treatment effects could be observed for the branching density or the length of the branching zone. Although the branching intensity was the only trait that was correlated with leaf N content (Additional file 6), the missing heritability exclude the integration of this trait into breeding schemes.

Genotypic differences were observed for all root traits except the maximal lateral root length in the first segment and the dry weight of embryonic roots. Accordingly, the heritability of the number and length of lateral roots was consistently moderate to high in both segments except for the maximal length in the first segment [h^2^ = 0 (low N); h^2^ = 0.41 (high N)] whereas the heritability was higher in the high N (0.41 ≤ h^2^ ≥ 0.72) than in the low N compartment (0.03 ≤ h^2^ ≥ 0.59) in general (Table 1b).

### Genotype-by-nitrogen interaction for lateral root characteristics

A significant genotype-by-N placement interaction was observed for the medium and maximum length of lateral roots. The ability of genotypes to form long first-order lateral roots and its genetic control is a very interesting research topic. Eventually such roots are able to replace some of the functions of their parental axile root, thus allowing a more flexible response of a root system.

### Relationship between root and shoot development

We found a strong connection between lateral root formation and shoot performance. We used correlations based on genotypic mean values to evaluate the dependencies between roots formed on the high and low N side and between roots and shoots. Root traits were strongly correlated among themselves (Fig. 4) and two major clusters of root traits could be identified by principal component analysis: the lateral root length and the number of lateral roots (Fig. 4). Interestingly, the number of laterals was closely correlated with the elongation of crown roots (Fig. 4) supporting the hypothesis that the number of laterals was mainly driven by axial root elongation rather than the branching density.

**Fig. 5:**
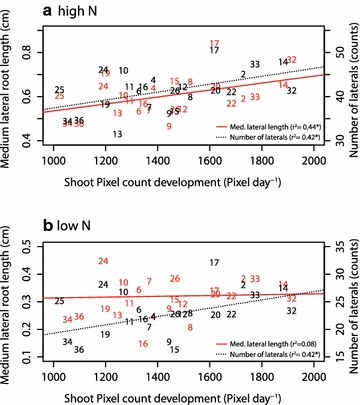
Correlation of shoot pixel count development (Pixel day^−1^) expressed as the slope best fitting the linear model and medium lateral root length (cm) or the number of lateral roots (counts) either under high N (**a**) or low N conditions (**b**). Significance levels: ≤0.001***, ≤0.01**, ≤0.05*. Correlation was done based on best linear unbiased predictors (BLUPS). The standard error of the difference was 323 pixels for the shoot and 0.1 cm for the lateral root length under high as well as under low N conditions

The leaf area was positively correlated to the medium length and number of lateral roots (Figs. 4, 5, Additional file 6) whereas the medium length was only under high N conditions correlated to shoot growth (Fig. 5). Lengths and densities of lateral roots measured in the second segment were stronger positively correlated with shoot traits than the first segment (Additional file 6). This inconsistency might be related to a feedback loop. N starvation during the establishment phase may have led to a limited link between shoot and root growth whereas in the second segment, the roots already profit from a higher N uptake resulting in a positive feedback from the shoot. This observation supports the hypothesis of de Kroon et al. [55] suggesting a coupling of nutrient sensing and coordinated growth of the root components and is in line with observations of in ’t Zandt et al. [29] demonstrating a dynamic differential response of crown and lateral root growth under either zero N or high N conditions. Furthermore, it indicates a signalling between roots and shoot transmitting the information about N availability that itself is transformed into differential growth e.g. by the provision of carbohydrates. Indeed, Tabata et al. [56] observed a signalling from the root to the shoot triggering the activity of nitrate transporters within root regions with high nitrate availability in *Arabidopsis thaliana.* An alternative explanation of this phenomenon could be a better N supply by the roots after the formation of laterals in the first segment resulting in an increasing root surface area and a simultaneously increased uptake of N after the change of the nutrient solution. Furthermore, a very high N stress level can lead to an inhibition of lateral root formation whereas a moderate stress can induce it [57].

### Considerations concerning a maize ideotype for improved foraging behaviour

There are many open questions with respect to the optimal root ideotype of maize. Foraging of N in nutrient-rich patches is mainly achieved by changing root system architecture. While, in general, the root length within the nutrient-rich patch increases, root growth outside the patch decreases [52, 53, 58, 59]. Thus, there is a trade-off between intensive foraging in patches and the overall direction of the root system to an available resource which might become important at critical stages of development, e.g. water and N at depth during grain filling. Accordingly, the proposed steep, cheap and deep ideotype to optimize water and N acquisition at depth to capture leached nitrate [7] includes “unresponsiveness of lateral branching to localized resource availability”. Paper-based systems offer a huge potential to establish rapid screens for N responsiveness.

This screening already provides an insight into the diversity of responses of genotypes towards inhomogeneous nutrient distributions and gives a strong indication that the responsiveness is closely linked to shoot development. However, further investigations with contrasting genotypes under field conditions are necessary, before drawing a conclusion on the utility of the presented screening method for selection purposes.

## Conclusion

The RADIX platform allowed studying dynamic changes in root-system architecture to split-root application of nitrogen. This is the first study evaluating such a differential response using a larger set of maize genotypes. A stronger selective root placement in the high N-compartment was related to an increased shoot development. This indicates that high early vigour might be related to a more intense foraging behaviour. In ongoing experiments, we aim to (1) verify these results under field conditions and (2) map the genomic regions controlling these responses using an association panel. Apart from studying nutrient-use efficiency, the system may be also used to evaluate responses to stresses like extreme pH, agrochemicals or microbes.

## Additional files

10.1186/s13007-016-0140-8 Constructional drawing of the RADIX platform (units:mm).
10.1186/s13007-016-0140-8 Constructional drawing of the imaging station (units:mm).
10.1186/s13007-016-0140-8 The video shows the eluation of the germination paper soacked in bromocresol green and afterwards watered with a basic (NH_4_HPO_4_) and an acidic (Ca(NO_3_)_2_) solution. A webcam was installed to image the elution. After 48 hours the bromocresol was washed out and the paper was sprayed with it again to visualize the pH gradient.
10.1186/s13007-016-0140-8 Shoot growth of 24 different genotypes quantified by the increase in number of pixels after the start of the split root treatment.
10.1186/s13007-016-0140-8 A) Best linear unbiased prediction (BLUPS) of parameters with significant treatment effect, but no genotype effect or genotype: treatment interaction. B) Prediction of mean values of parameters with significant genotype effect, but no genotype: treatment interaction. C) Prediction of mean values of parameters with significant genotype: treatment interaction. Abbreviations: Number of lateral roots in the first segment (No_Lat_ 1^st^) or second segment (No_Lat_ 2^nd^), length of representative lateral root in the first segment (Med_Lat_ 1^st^) or second segment (Med_Lat_ 2^nd^), maximal lateral root length in the first segment (Max_Lat_ 1^st^) or in the second segment (Max_Lat_ 2^nd^), elongation rate crown roots (ER_Cr_), length of crown roots at solution change (intercept; IC_Cr_), embryonic root dry weight (DW_ER_), crown root dry weight (DW_CR_), chlorophyll measurements (SPAD, measured leaf area (LA_m_), shoot dry weight (DW_S_), number of pixels specifying the leaf area (LA_Pix_), shoot pixel count at solution change (intercept; IC_S_), shoot pixel count development (ER_S_), N content in the leaf in % of total dry weight (N in %).
10.1186/s13007-016-0140-8 Correlation coefficients (Pearson’s r) of traits. Correlation was done based on best linear unbiased estimates (BLUPS). Abbreviations: Number of lateral roots in the first segment (No_Lat_ 1^st^) or second segment (No_Lat_ 2^nd^), length of representative lateral root in the first segment (Med_Lat_ 1^st^) or second segment (Med_Lat_ 2^nd^), maximal lateral root length in the first segment (Max_Lat_ 1^st^) or in the second segment (Max_Lat_ 2^nd^), branching density in the first segment (Br_Lat_1^st^) or in the second segment (Br_Lat_2^nd^), branching density across both segments (Br_Lat_Tot), length of the branching zone (LBrZone), total number of lateral roots (No_Lat_Tot), elongation rate crown roots (ER_Cr_), length of crown roots at solution change (intercept; IC_Cr_), embryonic root dry weight (DW_ER_), crown root dry weight (DW_CR_), leaf greenness (SPAD), leaf area measured (LA_m_), dry weight shoot (DWS), leaf area pixel based (LA_Pix_), shoot pixel count at solution change intercept (IC_S_), shoot pixel count development (ER_S_), N content in the leaf in % of total dry weight (N). Significance level: ≤ 0.001 ***, ≤ 0.01**, ≤ 0.05*.
10.1186/s13007-016-0140-8 Increase in crown root length of 24 genotypes under high nitrogen after the start of the split root treatment.
10.1186/s13007-016-0140-8 Increase in crown root length of 24 genotypes under low nitrogen after the start of the split root treatment.
10.1186/s13007-016-0140-8 Residual vs. fitted crown root length of multiple linear models to determine intercept and slope of crown root development after solution change.
10.1186/s13007-016-0140-8 Increase of lateral roots after solution change above the last formed lateral.

## Abbreviations

RSA: root system architecture
N: nitrogen
QTL: quantitative trait loci
