# Supplementary figures and images for: RADIX: rhizoslide platform allowing high throughput digital image analysis of root system expansion
Source: Plant Methods. 2016 Sep 5;12(1):40. doi: 10.1186/s13007-016-0140-8 (PMC5011878; doi:10.1186/s13007-016-0140-8)

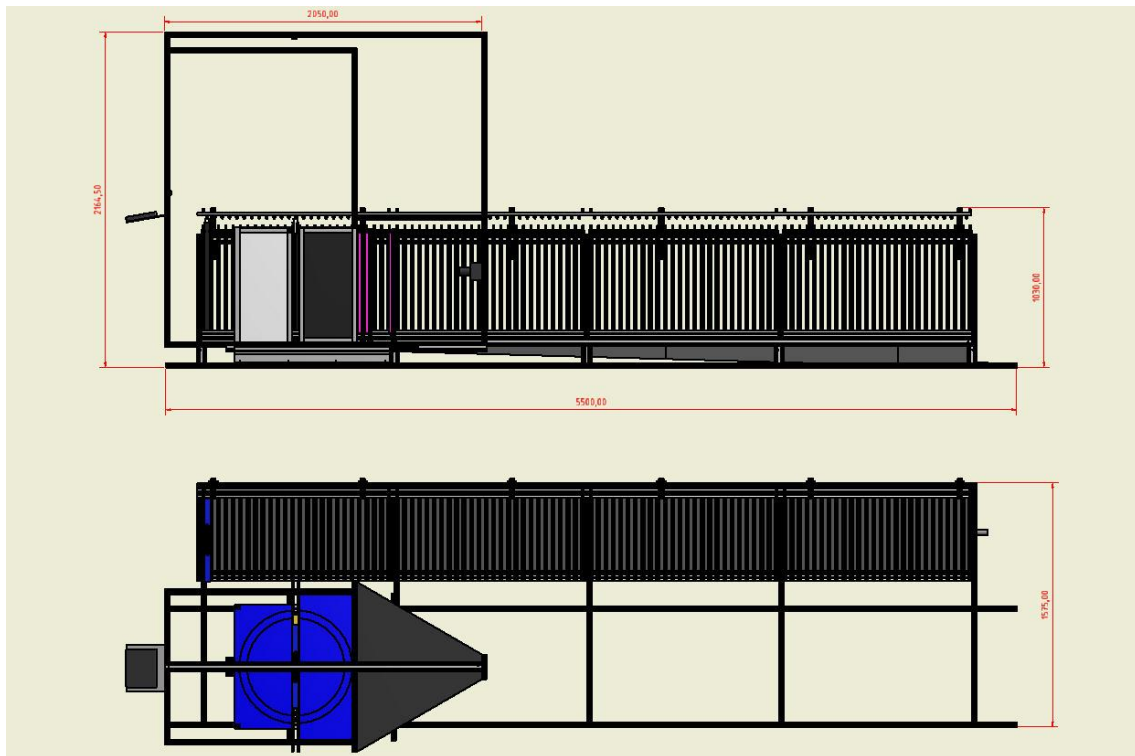

Additional file 1: Constructional drawing of the RADIX platform (units:mm).

Supplement: Supplementary file 1 — 10.1186/s13007-016-0140-8 Constructional drawing of the RADIX platform (units:mm). [file 13007_2016_140_MOESM1_ESM.pdf]

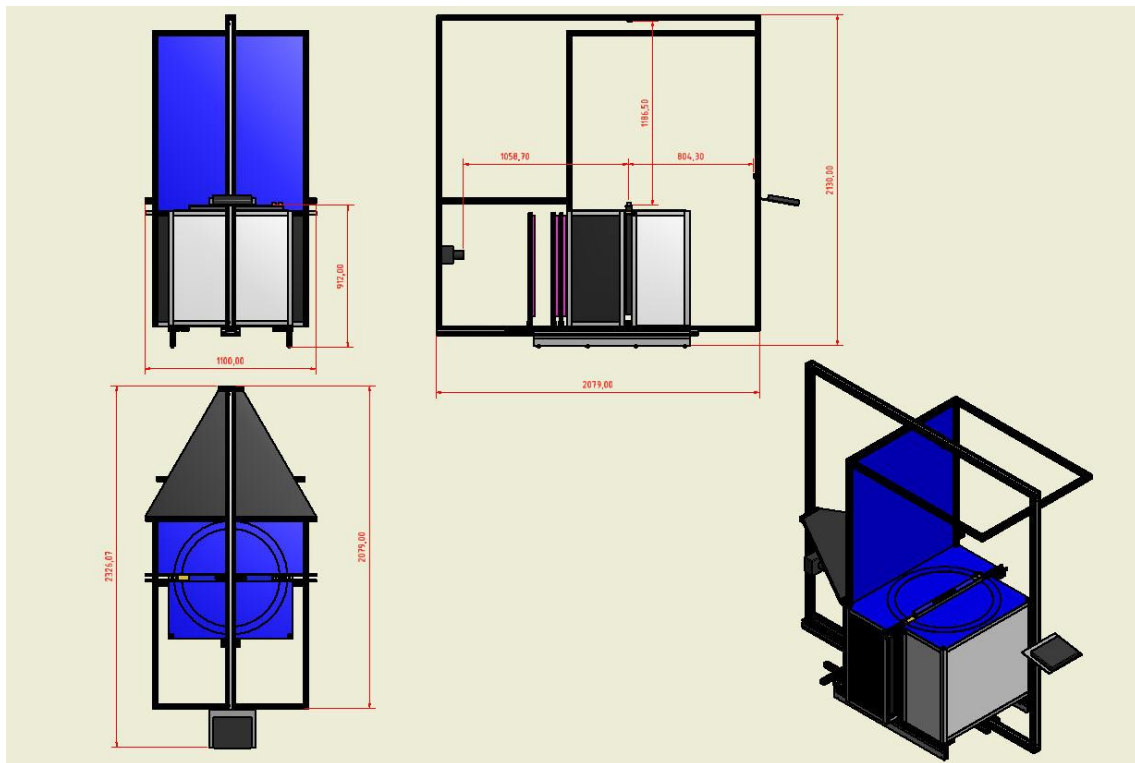

Additional file 2: Constructional drawing of the imaging station (units:mm).

Supplement: Supplementary file 2 — 10.1186/s13007-016-0140-8 Constructional drawing of the imaging station (units:mm). [file 13007_2016_140_MOESM2_ESM.pdf]
