# Supplementary material for: RADIX: rhizoslide platform allowing high throughput digital image analysis of root system expansion
Source: Plant Methods. 2016 Sep 5;12(1):40. doi: 10.1186/s13007-016-0140-8 (PMC5011878; doi:10.1186/s13007-016-0140-8)
Supplement: Supplementary file 5 — 10.1186/s13007-016-0140-8 A) Best linear unbiased prediction (BLUPS) of parameters with significant treatment effect, but no genotype effect or genotype: treatment interaction. B) Prediction of mean values of parameters with significant genotype effect, but no genotype: treatment interaction. C) Prediction of mean values of parameters with significant genotype: treatment interaction. Abbreviations: Number of lateral roots in the first segment (NoLat 1st) or second segment (NoLat 2nd), length of representative lateral root in the first segment (MedLat 1st) or second segment (MedLat 2nd), maximal lateral root length in the first segment (MaxLat 1st) or in the second segment (MaxLat 2nd), elongation rate crown roots (ERCr), length of crown roots at solution change (intercept; ICCr), embryonic root dry weight (DWER), crown root dry weight (DWCR), chlorophyll measurements (SPAD, measured leaf area (LAm), shoot dry weight (DWS), number of pixels specifying the leaf area (LAPix), shoot pixel count at solution change (intercept; ICS), shoot pixel count development (ERS), N content in the leaf in % of total dry weight (N in %). [file 13007_2016_140_MOESM5_ESM.pdf]

Additional file 5: A) Best linear unbiased prediction (BLUPS) of parameters with significant treatment effect, but no genotype effect or genotype: treatment interaction. B) Prediction of mean values of parameters with significant genotype effect, but no genotype: treatment interaction. C) Prediction of mean values of parameters with significant genotype: treatment interaction.

Abbreviations: Number of lateral roots in the first segment (No_{Lat} 1st) or second segment (No_{Lat} 2nd), length of representative lateral root in the first segment (Med_{Lat} 1st) or second segment (Med_{Lat} 2nd), maximal lateral root length in the first segment (Max_{Lat} 1st) or in the second segment (Max_{Lat} 2nd), elongation rate crown roots (ER_{Cr}), length of crown roots at solution change (intercept; IC_{Cr}), embryonic root dry weight (DW_{ER}), crown root dry weight (DW_{CR}), chlorophyll measurements (SPAD, measured leaf area (LA_m), shoot dry weight (DW_S), number of pixels specifying the leaf area (LA_{Pix}), shoot pixel count at solution change (intercept; IC_S), shoot pixel count development (ER_S), N content in the leaf in % of total dry weight (N in %).

A

**Treatment**	**Br_{Lat}1st** (lat cm^{-1})	**Br_{Lat}2nd** (lat cm^{-1})	**DW_{ER}** (mg)
**High**	2.12	1.81	28.76
**Low**	1.79	1.34	21.50
**HSD**	0.69	0.58	6.02
**avsed**	0.35	0.29	3.05

B

Genotype	Max_{Lat} 2nd (cm)	No_{Lat} 1st (count)	No_{Lat} 2nd (count)	IC_S (Pixel)	ER_S (cm^2)	LA_{Pix} (Pixel)	LA_m (cm)	DW_S (g)	N (%)
**A347**	1.86	49.70	32.41	1282	1914	22331	105.73	0.25	3.76
**B100**	1.70	42.83	37.18	1844	1191	13878	81.32	0.18	4.30
**B97**	1.24	30.79	25.52	1301	1434	16879	87.96	0.21	5.03
**EC169**	1.42	38.20	33.11	1220	1098	13360	76.01	0.18	4.31
**EZ11A**	1.84	37.17	32.53	1561	1383	16711	86.14	0.21	4.78
**EZ37**	1.87	48.24	36.39	925	1495	17240	87.45	0.19	4.03
**EZ47**	1.37	45.86	32.07	1097	1268	14662	81.34	0.19	4.40
**F1808**	1.35	37.08	26.11	1342	1344	15960	81.30	0.19	4.37
**F7028**	1.49	39.13	29.03	1337	1054	12401	71.75	0.17	4.15
**F98902**	1.41	38.04	32.47	1052	1368	16012	79.52	0.19	4.61
**FC1890**	1.38	31.13	28.70	1323	1328	15382	83.54	0.20	4.49
**FV353**	1.89	46.24	37.72	1118	1198	15339	82.39	0.20	4.02
**LAN496**	1.44	31.96	26.99	1998	1881	21352	107.32	0.27	3.81
**LH38**	1.71	43.78	36.63	1012	1444	16729	86.58	0.20	4.26
**Mo17**	1.32	29.43	22.23	1480	1729	20066	108.18	0.26	4.31
**MS153**	1.63	32.61	26.54	907	1245	14473	76.85	0.18	4.12
**MS71**	1.40	40.12	31.46	887	1465	16318	87.69	0.21	4.43
**N25**	1.62	36.24	32.12	1693	1525	18336	96.99	0.22	4.23
**N6**	2.02	41.67	33.40	1027	1466	16556	84.29	0.20	5.07
**Oh33**	1.87	50.03	41.37	1651	1023	12261	72.11	0.17	3.90
**(RootABA1-)**	1.67	33.23	31.97	1781	1617	19832	97.54	0.25	4.22
**(RootABA1+)**	2.16	39.13	31.86	1344	1683	19913	99.99	0.24	4.69
**PH207**	1.84	39.19	36.83	1717	1292	15623	85.17	0.20	3.81
**W64A**	1.77	32.71	32.06	527	1774	20952	95.12	0.23	3.61
**HSD**	**1.30**	**28.96**	**23.22**	**1883**	**1190**	**12738**	**67.64**	**0.17**	**1.56**
**avsed**	**0.35**	**7.79**	**6.24**	**499**	**315**	**3392**	**18.01**	**0.05**	**0.41**

C

Genotype	ER_{Cr}		$Med_{Lat}1^{st}$		$Med_{Lat}2^{nd}$		$Max_{Lat}1^{st}$		LBrZ		DW_{CR}	
	high	low	high	low	high	low	high	low	high	low	high	low
**A347**	1.66	1.15	1.11	0.38	0.77	0.31	2.58	1.14	76.29	72.11	35.94	21.70
**B100**	1.77	1.21	1.14	0.41	0.65	0.45	2.25	1.15	71.45	56.31	33.96	24.82
**B97**	1.62	1.17	1.07	0.35	0.48	0.25	1.88	0.60	60.48	52.06	33.08	19.78
**EC169**	1.75	1.08	1.04	0.32	0.64	0.25	2.12	0.72	72.73	63.88	39.62	17.06
**EZ11A**	1.75	1.09	1.07	0.35	0.54	0.29	2.44	1.24	76.94	54.72	41.89	18.67
**EZ37**	1.66	1.20	1.16	0.44	0.61	0.37	2.41	1.33	66.73	58.82	32.09	19.82
**EZ47**	1.55	1.21	1.09	0.37	0.57	0.15	2.13	0.61	70.66	59.57	34.27	15.79
**F1808**	1.60	1.14	1.05	0.33	0.48	0.24	1.98	0.71	68.43	58.92	32.26	19.03
**F7028**	1.59	1.21	1.06	0.34	0.54	0.37	2.07	0.91	68.26	55.84	30.76	18.77
**F98902**	1.61	1.31	1.08	0.36	0.53	0.34	2.01	0.81	62.27	61.54	32.68	26.43
**FC1890**	1.66	1.13	1.09	0.36	0.71	0.27	2.05	0.71	66.86	56.65	37.71	19.98
**FV353**	1.68	1.17	1.15	0.43	0.65	0.36	2.50	1.28	77.31	62.68	39.45	25.02
**LAN496**	1.44	1.22	1.04	0.32	0.47	0.28	1.99	0.88	70.24	63.71	35.90	25.64
**LH38**	1.58	1.28	1.08	0.36	0.59	0.39	2.28	1.13	63.22	57.39	29.92	32.98
**Mo17**	1.61	1.23	1.05	0.32	0.53	0.25	1.96	0.69	58.95	60.99	30.52	23.01
**MS153**	1.60	1.10	1.10	0.38	0.67	0.31	2.42	0.85	61.38	55.95	31.74	22.30
**MS71**	1.67	1.17	1.11	0.39	0.67	0.21	2.13	0.67	71.29	70.54	34.51	27.55
**N25**	1.65	1.16	1.07	0.35	0.54	0.39	2.14	1.10	63.03	47.30	34.75	21.90
**N6**	1.70	1.15	1.19	0.46	0.60	0.54	2.47	1.56	62.05	47.73	34.20	23.11
**Oh33**	1.73	1.19	1.19	0.46	0.84	0.34	2.55	1.19	81.21	67.94	40.78	27.84
**(RootABA1-)**	1.66	1.11	1.14	0.42	0.57	0.32	2.25	1.09	67.36	57.01	37.07	35.50
**(RootABA1+)**	1.46	1.14	1.21	0.49	0.58	0.30	2.75	1.56	78.19	59.13	34.75	28.95
**PH207**	1.75	1.06	1.09	0.37	0.60	0.38	2.38	1.30	75.47	63.35	38.90	18.88
**W64A**	1.55	1.11	1.11	0.39	0.65	0.33	2.42	1.11	64.55	54.72	41.46	21.80
**HSD**	0.94		0.52		0.58		1.80		61.37		35.59	
**avsed**	0.23		0.13		0.14		0.44		15.05		8.74	
