# Supplementary material for: RADIX: rhizoslide platform allowing high throughput digital image analysis of root system expansion
Source: Plant Methods. 2016 Sep 5;12(1):40. doi: 10.1186/s13007-016-0140-8 (PMC5011878; doi:10.1186/s13007-016-0140-8)
Supplement: Supplementary file 7 — 10.1186/s13007-016-0140-8 Increase in crown root length of 24 genotypes under high nitrogen after the start of the split root treatment. [file 13007_2016_140_MOESM7_ESM.pdf]

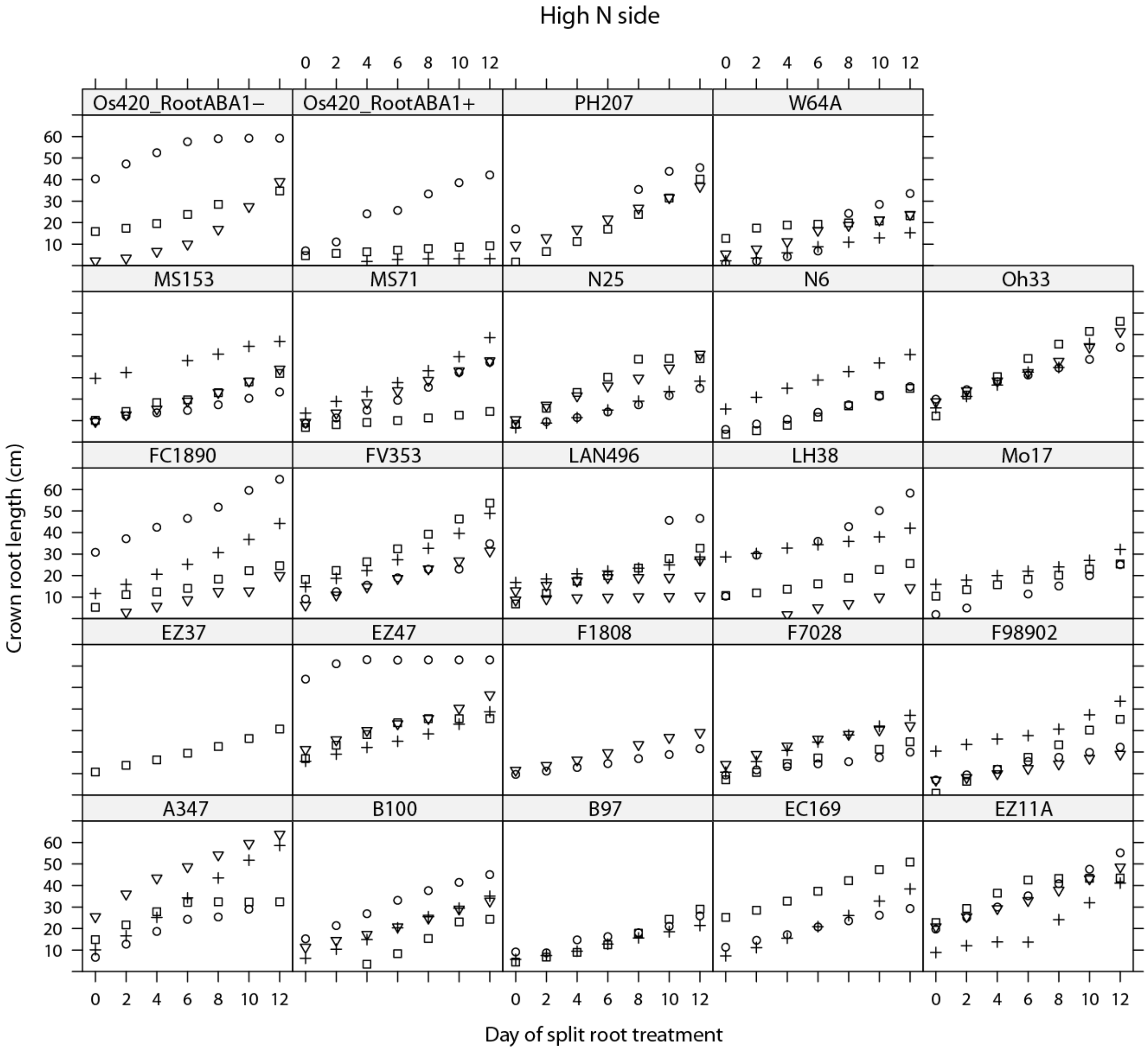

Additional file 7: Increase in crown root length of 24 genotypes under high nitrogen after the start of the split root treatment.
