# Supplementary material for: RADIX: rhizoslide platform allowing high throughput digital image analysis of root system expansion
Source: Plant Methods. 2016 Sep 5;12(1):40. doi: 10.1186/s13007-016-0140-8 (PMC5011878; doi:10.1186/s13007-016-0140-8)
Supplement: Supplementary file 9 — 10.1186/s13007-016-0140-8 Residual vs. fitted crown root length of multiple linear models to determine intercept and slope of crown root development after solution change. [file 13007_2016_140_MOESM9_ESM.pdf]

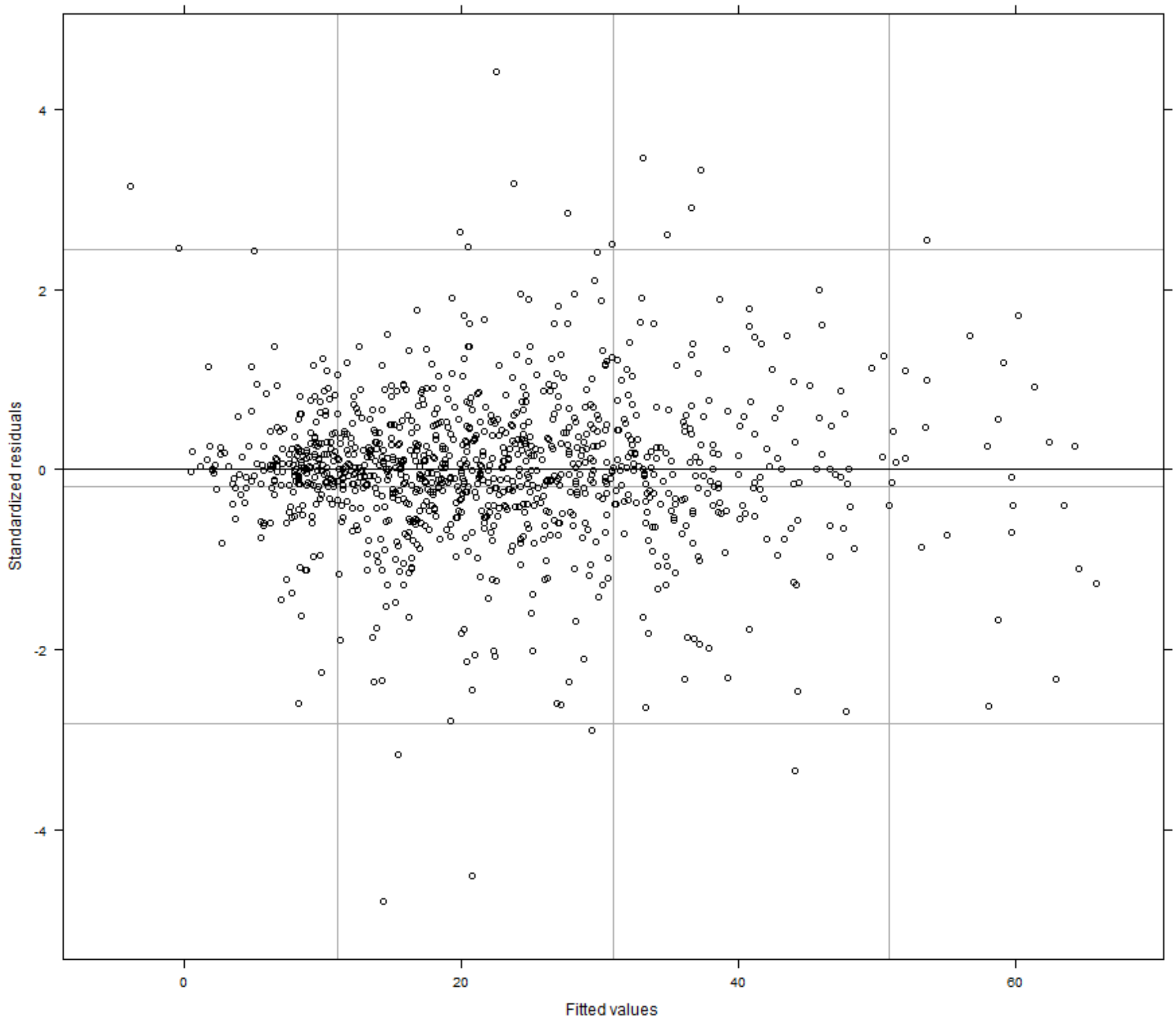

Additional file 9: Residual vs. fitted crown root length of multiple linear models to determine intercept and slope of crown root development after solution change.
