# Supplementary material for: RADIX: rhizoslide platform allowing high throughput digital image analysis of root system expansion
Source: Plant Methods. 2016 Sep 5;12(1):40. doi: 10.1186/s13007-016-0140-8 (PMC5011878; doi:10.1186/s13007-016-0140-8)
Supplement: Supplementary file 10 — 10.1186/s13007-016-0140-8 Increase of lateral roots after solution change above the last formed lateral. [file 13007_2016_140_MOESM10_ESM.pdf]

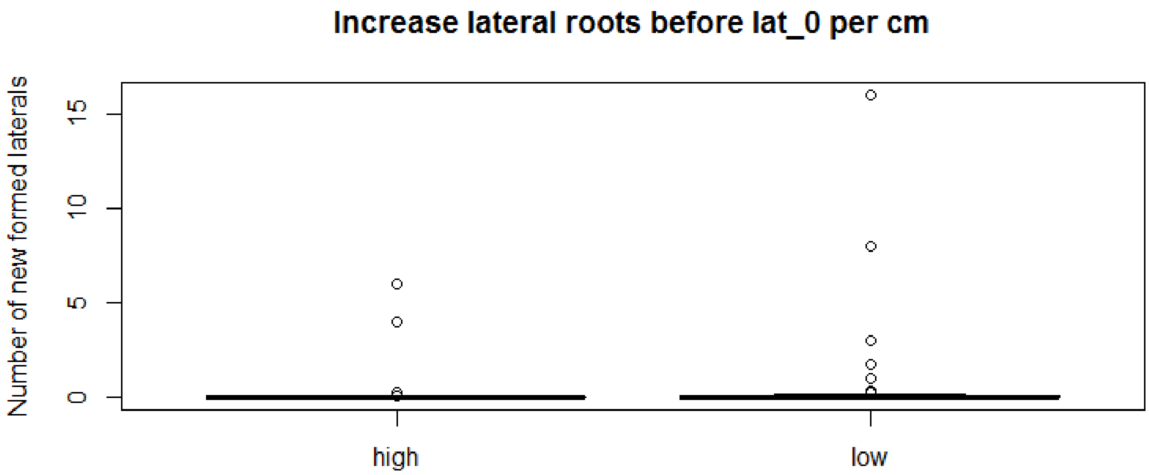

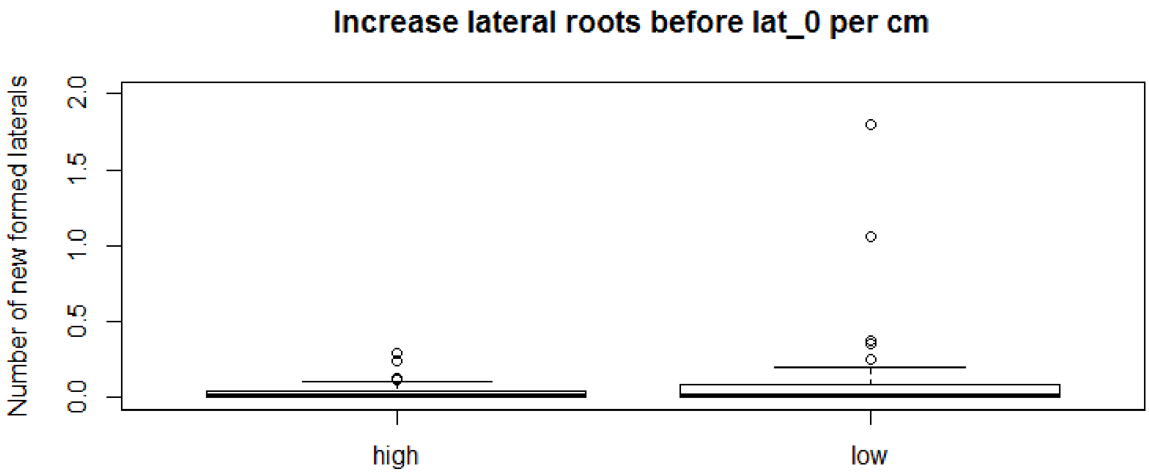

Additional file 10: Increase of lateral roots after solution change above the last formed lateral.
