# Supplementary material for: RADIX: rhizoslide platform allowing high throughput digital image analysis of root system expansion
Source: Plant Methods. 2016 Sep 5;12(1):40. doi: 10.1186/s13007-016-0140-8 (PMC5011878; doi:10.1186/s13007-016-0140-8)
Supplement: Supplementary file 6 — 10.1186/s13007-016-0140-8 Correlation coefficients (Pearson’s r) of traits. Correlation was done based on best linear unbiased estimates (BLUPS). Abbreviations: Number of lateral roots in the first segment (NoLat 1st) or second segment (NoLat 2nd), length of representative lateral root in the first segment (MedLat 1st) or second segment (MedLat 2nd), maximal lateral root length in the first segment (MaxLat 1st) or in the second segment (MaxLat 2nd), branching density in the first segment (BrLat1st) or in the second segment (BrLat2nd), branching density across both segments (BrLatTot), length of the branching zone (LBrZone), total number of lateral roots (NoLatTot), elongation rate crown roots (ERCr), length of crown roots at solution change (intercept; ICCr), embryonic root dry weight (DWER), crown root dry weight (DWCR), leaf greenness (SPAD), leaf area measured (LAm), dry weight shoot (DWS), leaf area pixel based (LAPix), shoot pixel count at solution change intercept (ICS), shoot pixel count development (ERS), N content in the leaf in % of total dry weight (N). Significance level: ≤ 0.001 ***, ≤ 0.01**, ≤ 0.05*. [file 13007_2016_140_MOESM6_ESM.pdf]

Additional file 6: Correlation coefficients (Pearson's r) of traits. Correlation was done based on best linear unbiased estimates (BLUPS). Abbreviations: Number of lateral roots in the first segment (No_{Lat} 1st) or second segment (No_{Lat} 2nd), length of representative lateral root in the first segment (Med_{Lat} 1st) or second segment (Med_{Lat} 2nd), maximal lateral root length in the first segment (Max_{Lat} 1st) or in the second segment (Max_{Lat} 2nd), branching density in the first segment ($Br_{Lat}1^{st}$) or in the second segment ($Br_{Lat}2^{nd}$), branching density across both segments ($Br_{Lat}Tot$), length of the branching zone (LBrZone), total number of lateral roots ($No_{Lat}Tot$), elongation rate crown roots (ER_{Cr}), length of crown roots at solution change (intercept; IC_{Cr}), embryonic root dry weight (DW_{ER}), crown root dry weight (DW_{CR}), leaf greenness (SPAD), leaf area measured (LA_m), dry weight shoot (DWS), leaf area pixel based (LA_{Pix}), shoot pixel count at solution change intercept (IC_S), shoot pixel count development (ER_S), N content in the leaf in % of total dry weight (N). Significance level: ≤ 0.001 ***, ≤ 0.01**, ≤ 0.05*.

Pearson's r	$No_{Lat}1^{st}$	$No_{Lat}2^{nd}$	$Med_{Lat}1^{st}$	$Med_{Lat}2^{nd}$	$Max_{Lat}1^{st}$	$Max_{Lat}2^{nd}$	$Br_{Lat}1^{st}$	$Br_{Lat}2^{nd}$	$Br_{Lat}Tot$	LBrZone	$No_{Lat}Tot$	ER_{CR}	IC_{CR}	DW_{ER}	DW_{CR}	SPAD	LA_m	DWS	LA_{Pix}	IC_S	ER_S	N
$No_{Lat}1^{st}$	1	0.78***	0.51*	0.51*	0.57**	0.51*	0.34*	0.36*	0.36*	0.58**	0.95***	0.47*	0.38*	0.30	0.05	-0.30	0.42*	0.46*	0.39	0.53**	0.39	0.05
$No_{Lat}2^{nd}$		1	0.58**	0.69***	0.70***	0.64***	0.23	0.37	0.30	0.45*	0.90***	0.49*	0.05	0.21	0.39	-0.24	0.52**	0.55**	0. 48*	0.46*	0.46*	0.16
$Med_{Lat}1^{st}$			1	0.62***	0.76***	0.77***	0.09	0.21	0.15	0.22	0.59**	0.08	-0.16	-0.12	0.42*	-0.36	0.21	0.26	0.12	0.56**	0.09	0.03
$Med_{Lat}2^{nd}$				1	0.68***	0.66***	0.43*	0.50*	0.47*	0.20	0.64**	0.45*	0.00	0.27	0.47*	-0.36	0.41*	0.45*	0.38	0.29	0.36	0.01
$Max_{Lat}1^{st}$					1	0.80***	0.40*	0.52**	0.46*	0.30	0.71***	0.16	-0.14	0.10	0.52**	-0.23	0.54**	0.55**	0.50*	0.33	0.46*	-0.06
$Max_{Lat}2^{nd}$						1	0.29	0.32	0.31	0.34	0.67***	0.03	0.06	0.21	0.45*	-0.52**	0.46*	0.46*	0.44*	0.28	0.44*	0.12
$Br_{Lat}1^{st}$							1	0.94***	0.99***	-0.15	0.38	0.35	0.02	0.30	0.06	-0.01	0.35	0.29	0.22	0.34	0.25	-0.48
$Br_{Lat}2^{nd}$								1	0.98***	-0.15	0.43*	0.39	-0.12	0.22	0.24	0.02	0.39	0.34	0.29	0.36	0.30	-0.38*
$Br_{Lat}Tot$									1	-0.17	0.40*	0.36	-0.05	0.27	0.14	0.00	0.37	0.31	0.25	0.35	0.27	-0.44**
LBrZone										1	0.55**	0.14	0.39	0.22	0.28	-0.37	0.45*	0.48*	0.52**	0.29	0.47*	0.19
NoLatTot											1	0.43*	0.22	0.28	0.19	-0.28	0.48*	0.51*	0.47*	0.44*	0.47*	0.12
ER_{CR}												1	0.17	0.11	0.04	-0.07	0.09	0.13	0.05	0.28	0.05	-0.09
IC_{CR}													1	0.26	-0.16	-0.47*	0.21	0.24	0.26	-0.09	0.26	0.23
DW_{ER}														1	0.08	0.02	0.56**	0.59**	0.58**	-0.05	0.61**	-0.16
DW_{CR}															1	-0.34	0.64***	0.65***	0.59**	0.35	0.55**	-0.01
SPAD																1	-0.22	-0.24	-0.28	-0.12	-0.30	-0.19
LA_m																	1	0.98***	0.95***	0.20	0.94***	-0.13
DWS																		1	0.93***	0.26	0.92***	-0.08
LA_{Pix}																			1	0.02	0.99***	0.03
IC_S																				1	-0.02	-0.22
ER_S																					1	-0.01
N																						1
